# Post-Embryonic Gonad Development in the Capital-Breeding Seed Beetle *Zabrotes subfasciatus* (Bruchinae): A Morphological View

**DOI:** 10.3390/insects17090938

**Published:** 2026-09-08

**Authors:** Juliana Ramos Martins, Márcia Maria Gentile Bitondi, Sílvia de Oliveira Miranda, Talita Sarah Mazzoni, Angel Roberto Barchuk

**Affiliations:** 1 Departamento de Biologia Celular e do Desenvolvimento, Instituto de Ciências Biomédicas, Universidade Federal de Alfenas (UNIFAL-MG), Alfenas CEP 37133-840, MG, Brazil; juliana.ramos@unifal-mg.edu.br (J.R.M.); silvia.miranda@sou.unifal-mg.edu.br (S.d.O.M.); talita.mazzoni@unifal-mg.edu.br (T.S.M.); 2 Departamento de Biologia, Faculdade de Filosofia, Ciências e Letras de Ribeirão Preto, Universidade de São Paulo (USP), Ribeirão Preto CEP 14049-900, SP, Brazil; mmgbit@usp.br

**Keywords:** ovary, ovariole, testis, seed beetle, development

## Abstract

Post-embryonic gonad development determines when insects acquire reproductive competence, yet this process remains poorly characterized in Bruchinae seed beetles. Here, we examined ovarian and testicular development in the capital-breeding bean weevil *Zabrotes subfasciatus*, from larval stages to four-day-old adults, using stereomicroscopy, light microscopy, and scanning electron microscopy. Female gonads were already recognizable in larvae as ovaries composed of three ovarioles, each dominated by a proliferative germarial region and posterior duct-like domains. During pharate-pupal and pupal development, these structures remained largely previtellogenic but showed ductal integration associated with future calyx formation. The most extensive ovarian reorganization occurred in pharate adults, when terminal filaments, individualized oocytes, follicular cells, trophic cords, and the germarium–vitellarium transition became evident; acidophilic inclusions consistent with early yolk deposition further indicate that vitellogenesis begins before emergence. Male gonads also differentiated early, with larval testes containing germ stem cells, spermatogonia, spermatocytes, spermatids, cyst cells, and the internal portion of the efferent ducts. From the pharate-pupal stage onward, spermiogenesis intensified, and four-day-old adults displayed a fully organized reproductive tract. Together, these findings show that gonad development begins during larval life but attains functional organization mainly during late metamorphosis, supporting rapid reproductive readiness in this capital-breeding pest.

## Simple Summary

Seed beetles are important pests of stored legumes, but little is known about how their reproductive organs develop before adulthood. This study examined the formation of ovaries and testes in *Zabrotes subfasciatus*, a bean weevil whose adults depend mainly on reserves accumulated while feeding inside seeds as larvae. Using stereomicroscopy, histology, and scanning electron microscopy, we found that gonad development starts during larval life but that the main changes leading to reproductive readiness occur during late metamorphosis, especially between the pupal and pharate-adult stages. In females, this period includes the organization of the ovaries, the appearance of developing oocytes, follicular cells, trophic cords, and the first signs of yolk deposition, showing that egg maturation begins before adult emergence. In males, the testes also mature early, with advanced sperm development already evident before emergence. These results help explain why newly emerged adults of this pest can become reproductively active quickly and provide useful information for future studies on reproductive biology and pest-management strategies.

## 1. Introduction

Seed beetles (Coleoptera: Chrysomelidae: Bruchinae) are phytophagous insects strongly associated with leguminous plants (Fabaceae). Their larvae develop endophagously inside seeds, where they complete most of their growth while consuming cotyledonary and embryonic tissues. This seed-confined development underlies the economic importance of many Bruchinae as pests of field-grown and stored grains and also makes the group a useful model for studying how specialized life histories shape developmental and reproductive strategies [1,2].

Despite extensive information on host use, oviposition behaviour, taxonomy, and life-history variation in Bruchinae [1,3], the post-embryonic development of their reproductive organs remains poorly characterized. Developmental timing has been described for some seed beetles, including *Callosobruchus maculatus* and *Acanthoscelides macrophthalmus*, but detailed comparative accounts of ovarian and testicular differentiation are still scarce [4,5]. This gap is important because gonad development determines when newly emerged adults become reproductively competent, a particularly relevant issue for seed beetles whose adult stage may be short and only weakly dependent on feeding. The relationship between nutrition and reproduction provides a central framework for interpreting this developmental timing. In income-breeding insects, reproductive maturation is closely linked to nutrients acquired during adult life, as demonstrated in the beetles *Tenebrio molitor* [6] and *Nicrophorus* spp. [7]. In contrast, capital breeders rely mainly on reserves accumulated before reproduction, so gonadal maturation is expected to begin earlier and to progress during late juvenile or metamorphic stages [8]. Many Bruchinae fit this latter strategy because adults often feed little or not at all, and their reproductive output depends largely on larval resources. Accordingly, gonadal maturation in these beetles may be advanced, compressed, or coordinated with metamorphosis rather than delayed until after emergence [1,9].

*Zabrotes subfasciatus* [10] is a bruchine seed beetle native to Central and South America and now widely distributed in tropical bean-growing regions [11,12,13]. Females lay eggs on the seed surface, larvae bore into the seed after hatching, and development proceeds internally until pupation and adult emergence. Under favourable laboratory conditions, adults usually emerge about 26 days after oviposition at 30–35 °C [14]. Adult feeding and water intake appear to be facultative, reinforcing the view that this species depends primarily on reserves accumulated during larval development to support reproduction [1,3,15,16].

Previous work on *Z. subfasciatus* has shown that ovarian activation depends on both host seeds and male presence and that vitellogenesis begins before or soon after adult emergence [9]. However, the sequence by which larval gonadal primordia are transformed into functional adult ovaries and testes remains unknown. Addressing this question is relevant for three reasons. First, it clarifies how reproductive organ ontogeny proceeds in an insect whose immature development is spatially and nutritionally constrained inside a seed. Second, because many Bruchinae are economically important pests, understanding the timing of gonadal maturation may help explain their rapid reproductive potential and inform future pest-management approaches [17]. Third, it remains uncertain whether seed beetles follow the generalized gonadal developmental patterns described for other holometabolous beetles or whether their capital-breeding ecology is associated with modifications in the timing and organization of gonad development.

Based on the reproductive biology of *Z. subfasciatus* and on previous evidence that adult females possess three telotrophic meroistic ovarioles with early vitellogenic activity [9], we hypothesised that gonadal primordia are established during larval life and that maturation progresses through metamorphosis, allowing both ovaries and testes to approach functional readiness by adult emergence. Accordingly, this study aimed to describe the morphological and histological progression of gonad development in *Z. subfasciatus* from larval to adult stages, with emphasis on germ-cell proliferation, somatic differentiation, and the onset of gametogenic organization in both sexes.

## 2. Materials and Methods

### 2.1. Beetle Husbandry and Sampling

The *Z. subfasciatus* individuals used in this study originated from a stock population of approximately 6000 individuals, initially collected from bean seeds in Ribeirão Preto, São Paulo, Brazil, and maintained on bean (*Phaseolus vulgaris*, “carioquinha” variety) seeds in the laboratory since 1997. According to Souza et al. [18], Brazilian populations of *Z. subfasciatus* exhibit low genetic differentiation and weak geographic structure. To minimize potential inbreeding effects, individuals collected from bean seeds in Poços de Caldas and Alfenas, Minas Gerais, Brazil, have been incorporated into the stock population approximately every three months since 2007. The colony was maintained on bean seeds at 22 ± 5 °C and 70 ± 15% relative humidity, under dark conditions [14,16]. Additional information on the biology of this species is available in Bondar [19], Teixeira et al. [20], Teixeira and Gris [21], Teixeira and Zucoloto [22], and Cruz et al. [2].

**Figure 1:**
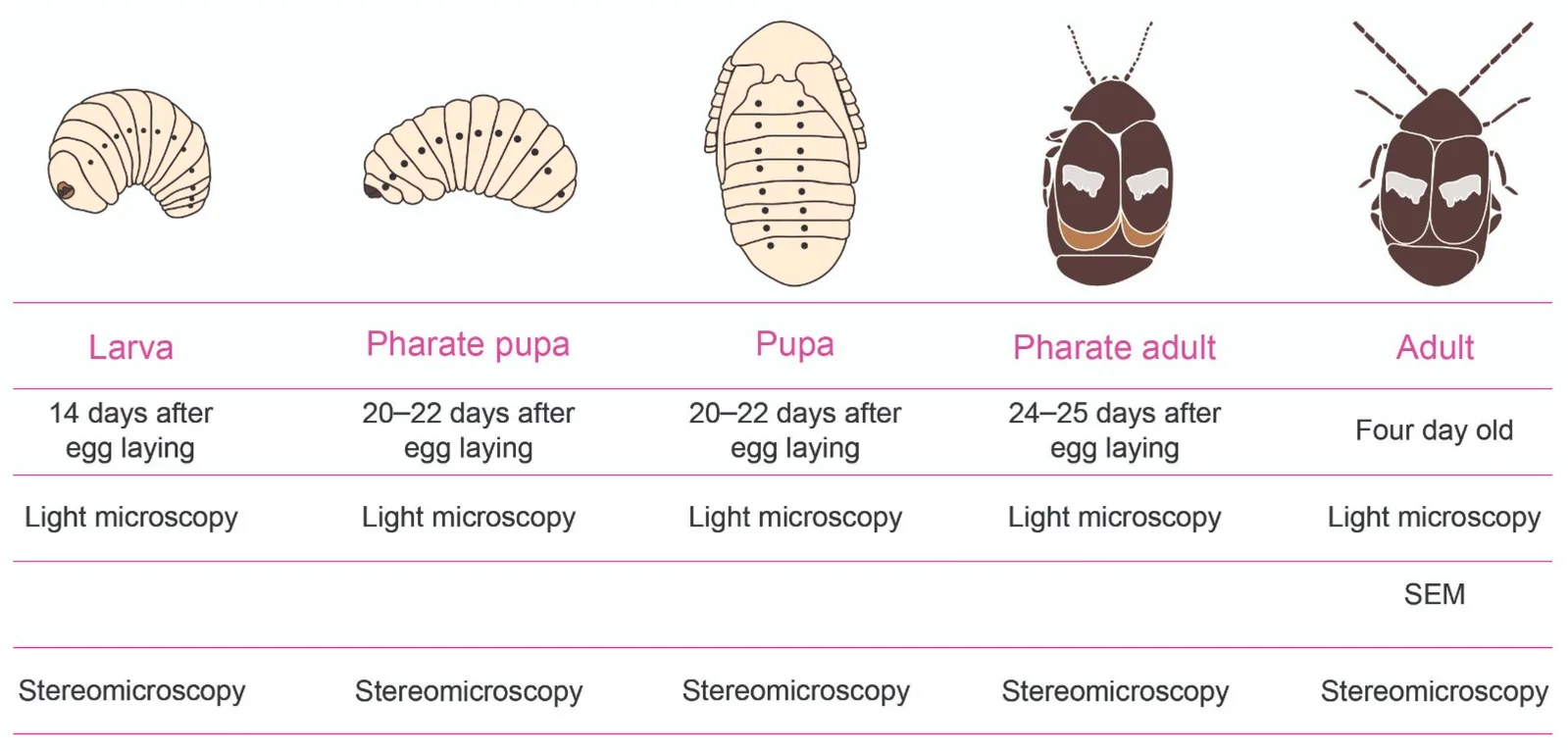
Experimental design used to study post-embryonic gonad development in *Zabrotes subfasciatus*. The scheme summarizes the sampled developmental stages and the microscopy approaches used for whole individuals and dissected gonads.

Larvae (14 days after egg laying), pharate pupae (20–22 days), pupae displaying white bodies (20–22 days), and pharate adults exhibiting dark pigmentation (24–25 days) (Figure 1) were carefully extracted from the seeds using a scalpel for cutting and No. 5 watchmaker’s forceps for insect removal. Adults were obtained from a population maintained in beans. For dissection, individuals were placed in a watch glass containing Phosphate-Buffered Saline (PBS) (137 mM NaCl, 2.7 mM KCl, 10 mM dibasic Na_2_HPO_4_, 1.8 mM monobasic KH_2_PO_4_; pH 7.2). Using No. 5 watchmaker’s forceps, No. 2 entomological pins, and a Stemi 305 stereomicroscope (Zeiss, Carl Zeiss, Jena, Germany), the abdominal cuticle was opened and internal tissues were removed. The ovaries and testes of developing and adult beetles were isolated from surrounding tissues and subsequently fixed. Whole individuals and dissected gonads were photographed using a stereomicroscope equipped with an Axiocam 105 color camera (Carl Zeiss, Jena, Germany) and ZEN 3.7 software. The light microscope images were captured using an Olympus CX21 microscope (Olympus Corporation, Tokyo, Japan) equipped with an AmScope MU1003 camera (AmScope 10MP aptina color CMOS, Ningbo, China).

### 2.2. Histology

Whole individuals were processed according to the protocol of Lussier et al. [23], with some modifications. Individuals were fixed at 4 °C in Schaffer’s fixative (37% formaldehyde and 90% ethanol, 1:2 ratio) supplemented with a calcium thioglycolate-based depilatory cream (DepiRoll Resistance^®^, BBP Cosméticos Ltda, Vinhedo, SP, Brazil; fixative:cream ratio, 2:1) and maintained in the fixative for 5 days at 4 °C. The samples were then washed in PBS, small holes were made in the cuticle using an entomological pin, and they were incubated in a modified Alcohol-Formalin-Acetic acid Fixative [24] (12 parts 80% ethanol, 4 parts 37% formaldehyde, and 1 part glacial acetic acid) for two days at 4 °C. Dissected ovaries and testes were fixed at 4 °C according to Karnovsky [25] for 24 h. After fixation, samples were dehydrated in ethanol (70%, 80%, 90%, and 95%, each for two 30-min intervals), and embedded in glycol methacrylate (Leica Historesin, Leica Biosystems, Nussloch, Germany) using a stepwise infiltration process, as follows: 2 parts 95% ethanol and 1 part Historesin infiltration solution for 1 h; 1 part 95% ethanol and 1 part Historesin infiltration solution for 1 h; 1 part 95% ethanol and 2 parts Historesin infiltration solution for 1 h; only Historesin infiltration solution for 2 days at 4 °C and 3 days at room temperature. The samples were sectioned to a thickness of 5 µm with a Leica Rotary Microtome (Leica Biosystems, Nussloch, Germany) and all sections were stained with Harris hematoxylin and eosin [26] (H&E). Micrographs of the sections were taken using a Nikon Eclipse 80i microscope (Nikon, Otawara, Japan) equipped with a digital camera and Nis-Element 3.1 imaging software.

### 2.3. Scanning Electron Microscopy (SEM)

Dissected adult ovaries and testes were fixed in Karnovsky’s solution for 24 h at 4 °C. Samples were then washed twice with PBS and post-fixed in 1% osmium tetroxide for 1 h. For removal of osmium remnants, samples were again washed twice with PBS and once with deionized water, each for a period of 10 min. Then a serial dehydration was done with increasing concentrations of ethanol (10, 30, 50, 70, 90, 100%). Each ethanol bath was done twice, for 10 min. Thereafter, samples were dried using a CO_2_ critical point drier (Tousimis Autosamdri^®^-815, Tousimis Research Corporation, Rockville, MD, USA). Specimens were finally coated with a gold sputter coater (Denton Vacuum, Denton Vacuum Enabling Innovation, Moorestown, NJ, USA) for a coat of 20 nm. Coated samples were observed and photographed using a scanning electron microscope (TESCAN-MIRA4 FEG, TESCAN, Brno, Czech Republic) using an Everhart-Thornley Secondary Electrons Detector and an In-Beam Secondary Electrons Detector (Denton Vacuum Enabling Innovation, USA) at 10 kV. Images were captured at the Centro de Microscopia, Universidade Federal de Alfenas, Minas Gerais, Brazil.

## 3. Results

### 3.1. General External Morphology of the Developing Gonads of Zabrotes subfasciatus

**Figure 2:**
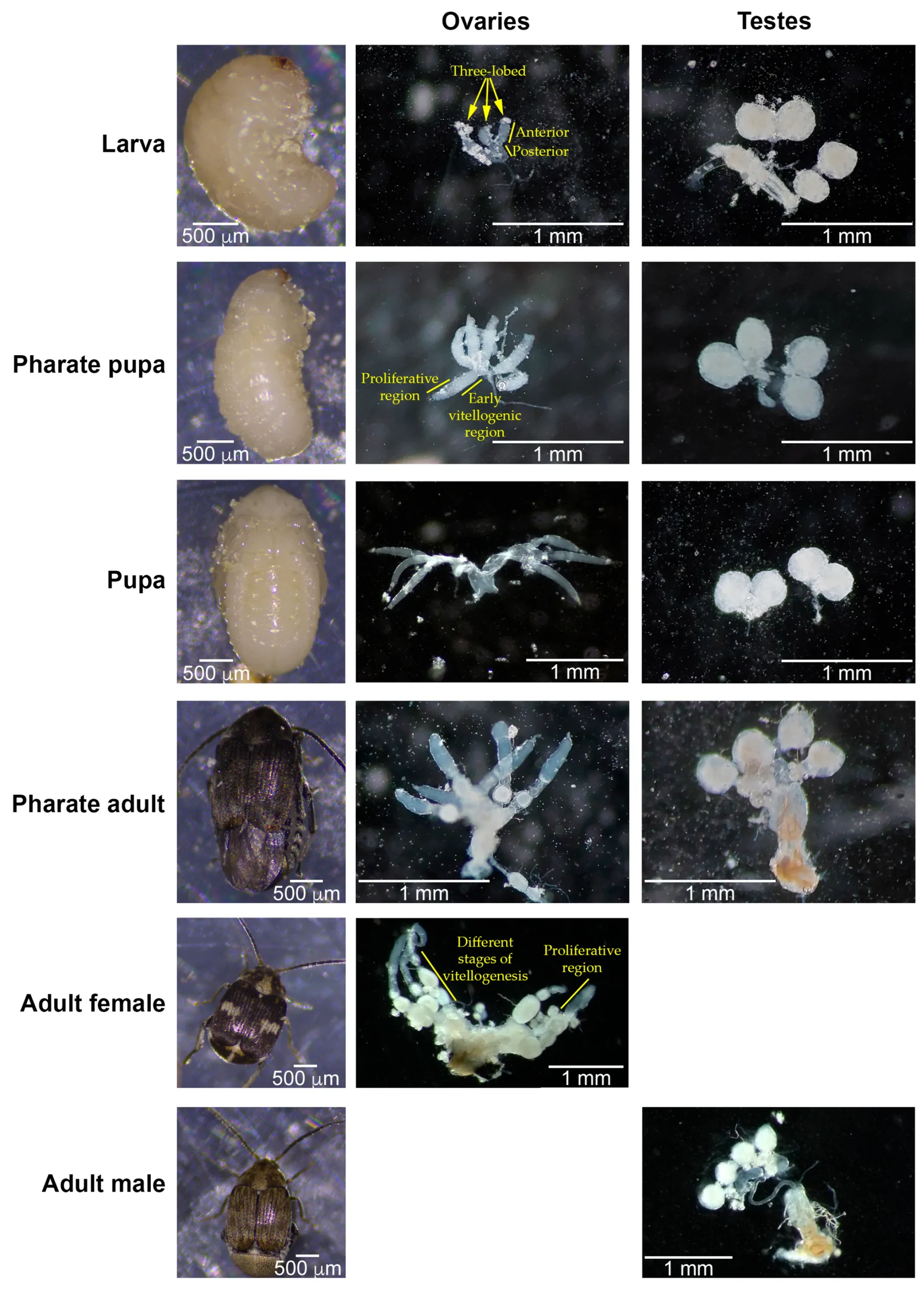
Anatomy of ovary and testis across post-embryonic developmental stages of *Zabrotes subfasciatus*. Larva, 14 days after egg laying; pharate pupa, 20–22 days; pupa, 20–22 days; pharate adult, 24–25 days. Stereomicroscope images of whole individuals (left column) and dissected gonads (remaining columns). The larval stage is represented by a single ovary, whereas both ovaries are shown at the remaining developmental stages. In the ovaries, “anterior” and “posterior” refer to the distal and proximal regions, respectively, as indicated in the image of the larval ovary.

Under the stereomicroscope, the ovary of a 14-day-old larva appears as a small, three-lobed, opaque structure formed by the ovarioles, which converge posteriorly at their proximal ends. In each ovariole, the distal region is slightly broader than the proximal portion, a pattern that becomes more evident in the subsequent pharate-pupal stage (Figure 2, middle column). From the pupal stage onward, the ovaries become more elongated and mostly translucent, although distinct functional domains are still not clearly visible in pupae. Such compartmentalization becomes evident only in pharate adults, in which the ovarioles can already be distinguished into proliferative and early vitellogenic regions, even though the distal portion remains relatively translucent. In adults, this organization is fully evident, with a clear separation between the proliferative region and the different stages of vitellogenesis.

Under the stereomicroscope, the testes of a 14-day larva appear larger than the ovaries, forming two paired opaque spheres (testicular follicles) connected by thin strands of tissue (Figure 2, right column). This general appearance is maintained through the pupal stage. In pharate adults, however, the testes are already connected to a well-developed proximal tract, similar to that observed in adults.

### 3.2. Histological Organization of the Larval Ovary of Zabrotes subfasciatus

**Figure 3:**
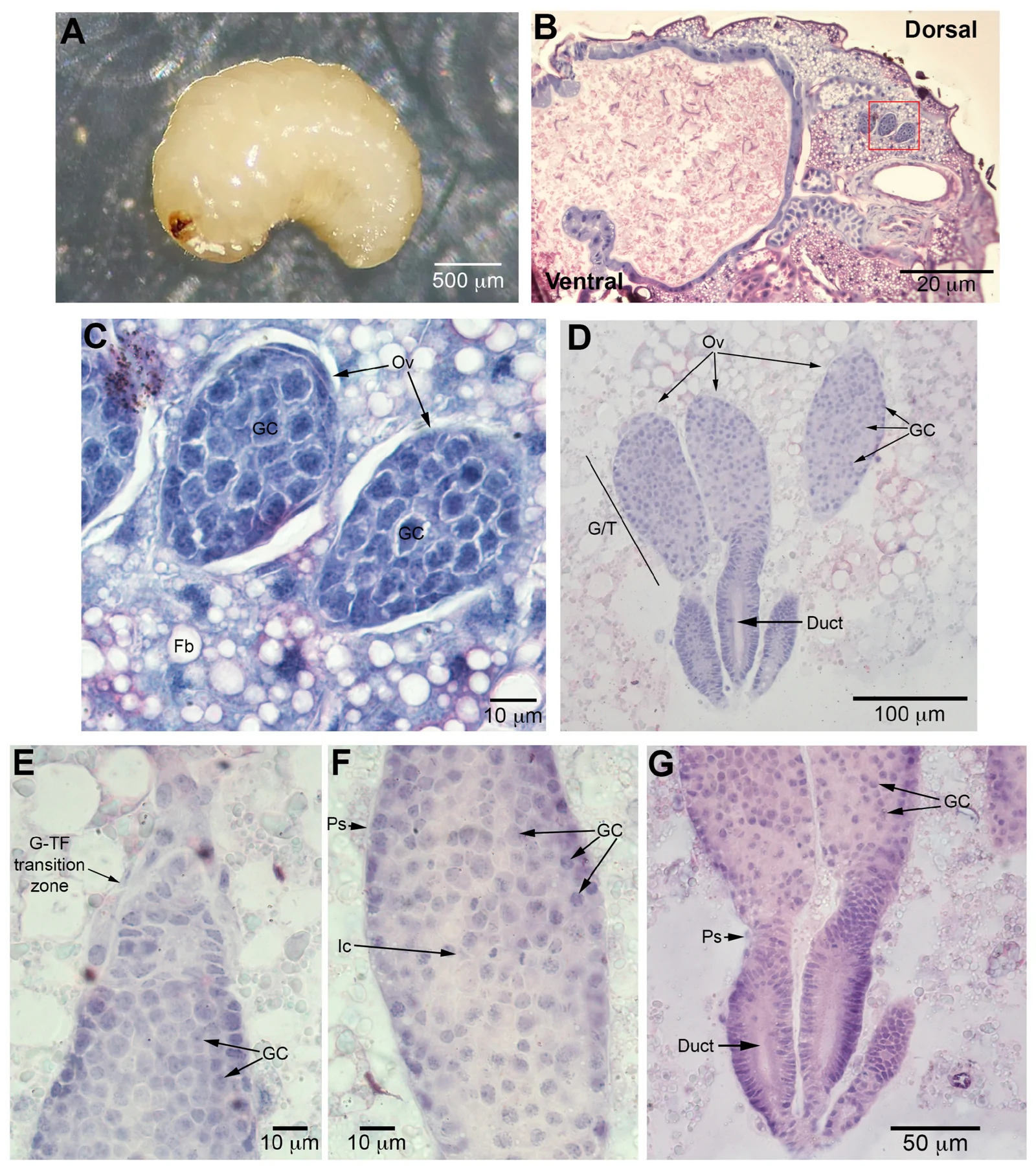
Morphology of the larval (14 days after egg laying) ovary of *Zabrotes subfasciatus*. (**A**) Stereomicroscope image of a female larva. (**B**–**G**) Light microscope images. (**B**) Longitudinal section of a larval body highlighting the anatomical location of the ovary (red label). (**C**–**G**) magnified views of distinct sections from the red-labelled area indicated in (**B**). Ov, ovariole; GC, germ cells; Fb, fat body cells; G/T, germarium/tropharium; G-TF, germarium/terminal filament; Ps, peritoneal sheath; Ic, interstitial cell.

Light microscopy revealed that the ovaries of *Z. subfasciatus* larvae (14 days after oviposition) are in the posterior-dorsal region of the abdomen and are surrounded by fat body tissue (Figure 3A–C). The ovarioles are differentiated into two main regions: a larger distal (anterior) region, the germarium, and a proximal (posterior) duct-like region (Figure 3D,G). These regions display distinct histomorphologies consistent with a germarium and an epithelial domain. The germarium is densely populated with germ cells interspersed with somatic interstitial cells and is enclosed by a peritoneal sheath (Figure 3D–G). The germarium continues into a transition zone to the terminal filament (Figure 3E), whereas proximately, it extends into the duct-like region, which is composed of epithelial-like cells (Figure 3D,G). The duct-like regions of the three ovarioles converge and appear to contribute to the final organization of the calyx.

**Figure 4:**
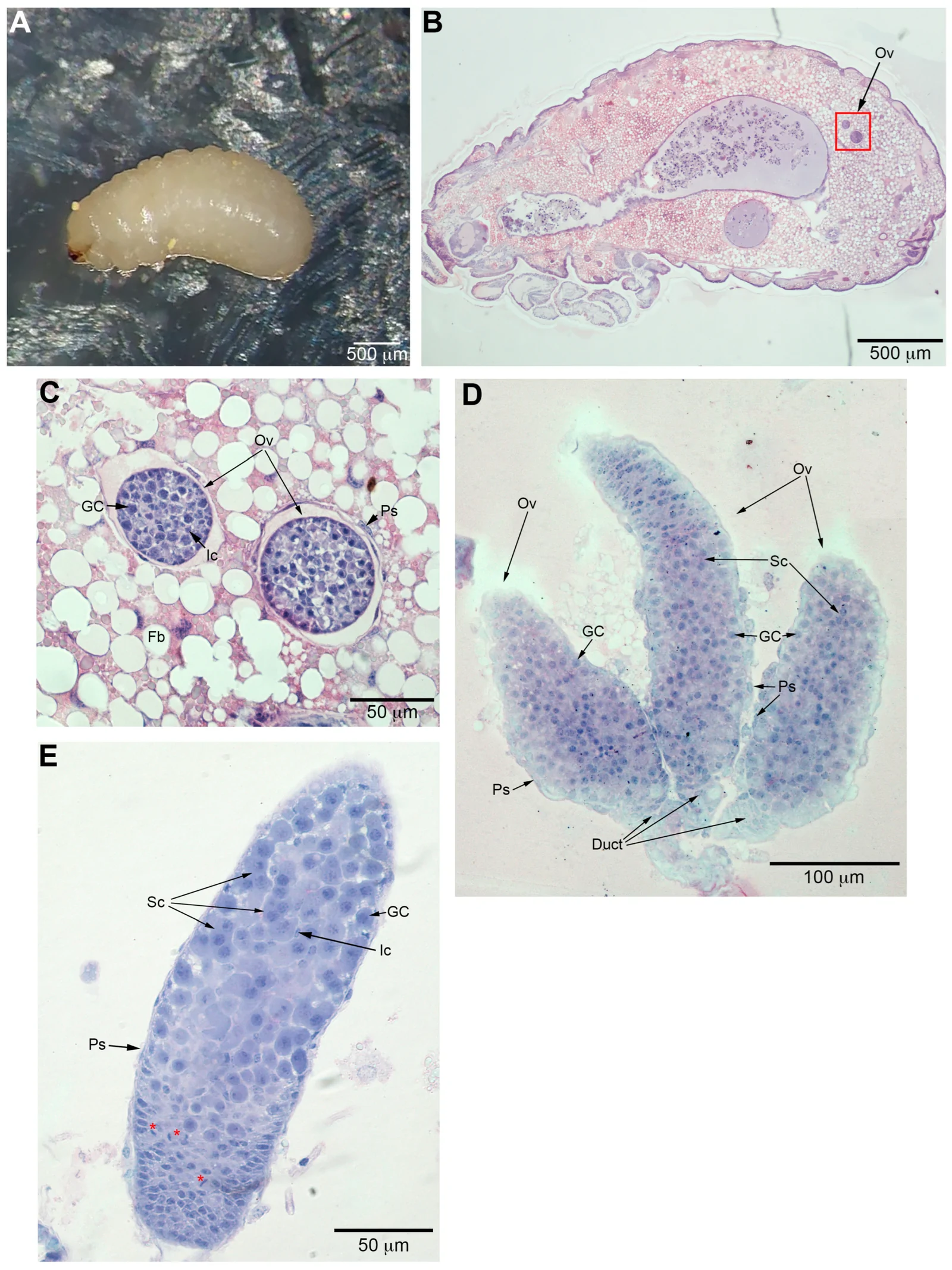
Morphology of the pharate-pupal (pre-pupa; 20–22 days after egg laying) ovary of *Zabrotes subfasciatus*. (**A**) Stereomicroscope image of a female pharate pupa. (**B**–**E**) Light microscope images. (**B**) Longitudinal section of a pharate-pupal body highlighting the anatomical location of the ovary (red label). (**C**) Transverse section of two ovarioles. (**D**,**E**) Longitudinal sections of dissected ovary (**D**) and ovariole (**E**). Ov, ovarioles; GC, germ cells; Fb, fat body cells; Ps, peritoneal sheath; Ic, interstitial cell; Sc, syncytial cells; *, dividing cells.

The organization of the pharate-pupal ovary (pre-pupa; 20–22 days after egg laying) remains broadly similar to that observed in 14-day-old larvae (Figure 4), although the germarium is noticeably enlarged at this stage (compare Figure 3D and Figure 4D) and contains syncytial cells (Figure 4D,E). In addition, the proximal duct-like regions of the three ovarioles, which contain mitotically active cells (Figure 4E), converge and extend into a single duct-like structure that is consistent with a calyx primordium; continuity to the adult calyx was not directly demonstrated. (Figure 4D). Thus, although the ovary still lacks the distinct compartmentalization observed in pharate adults, the pharate-pupal stage already exhibits structural rearrangements indicative of the early stages of ovariole maturation.

### 3.3. Histological Organization of the Pupal and Pharate-Adult Ovary of Zabrotes subfasciatus

**Figure 5:**
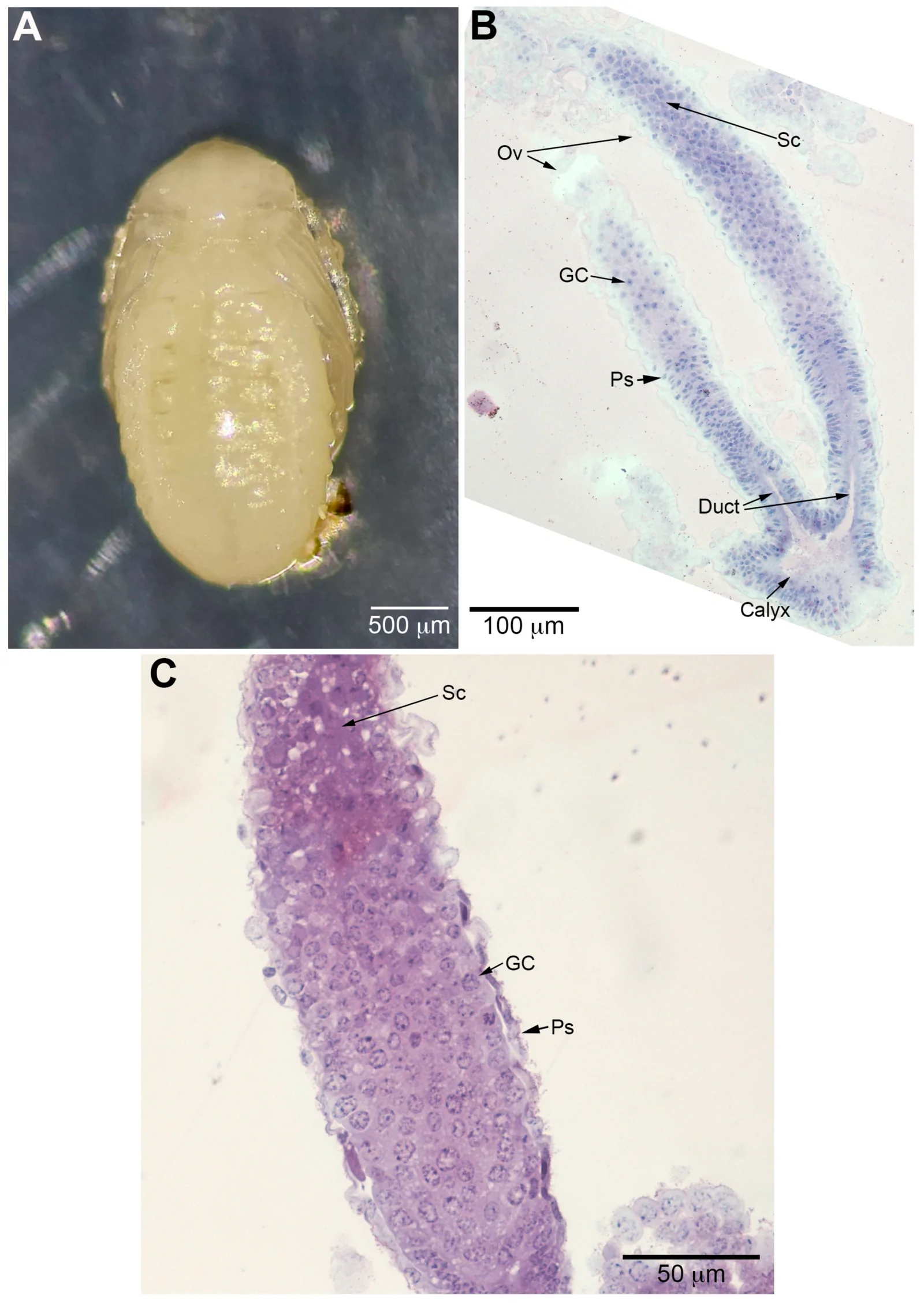
Morphology of the pupal (20–22 days after egg laying) ovary of *Zabrotes subfasciatus*. (**A**) Stereomicroscope image of a female pupa. Light microscope images of a dissected ovary (**B**) and an ovariole (**C**). Ov, ovarioles; Sc, syncytial cells; GC, germ cells; Ps, peritoneal sheath.

**Figure 6:**
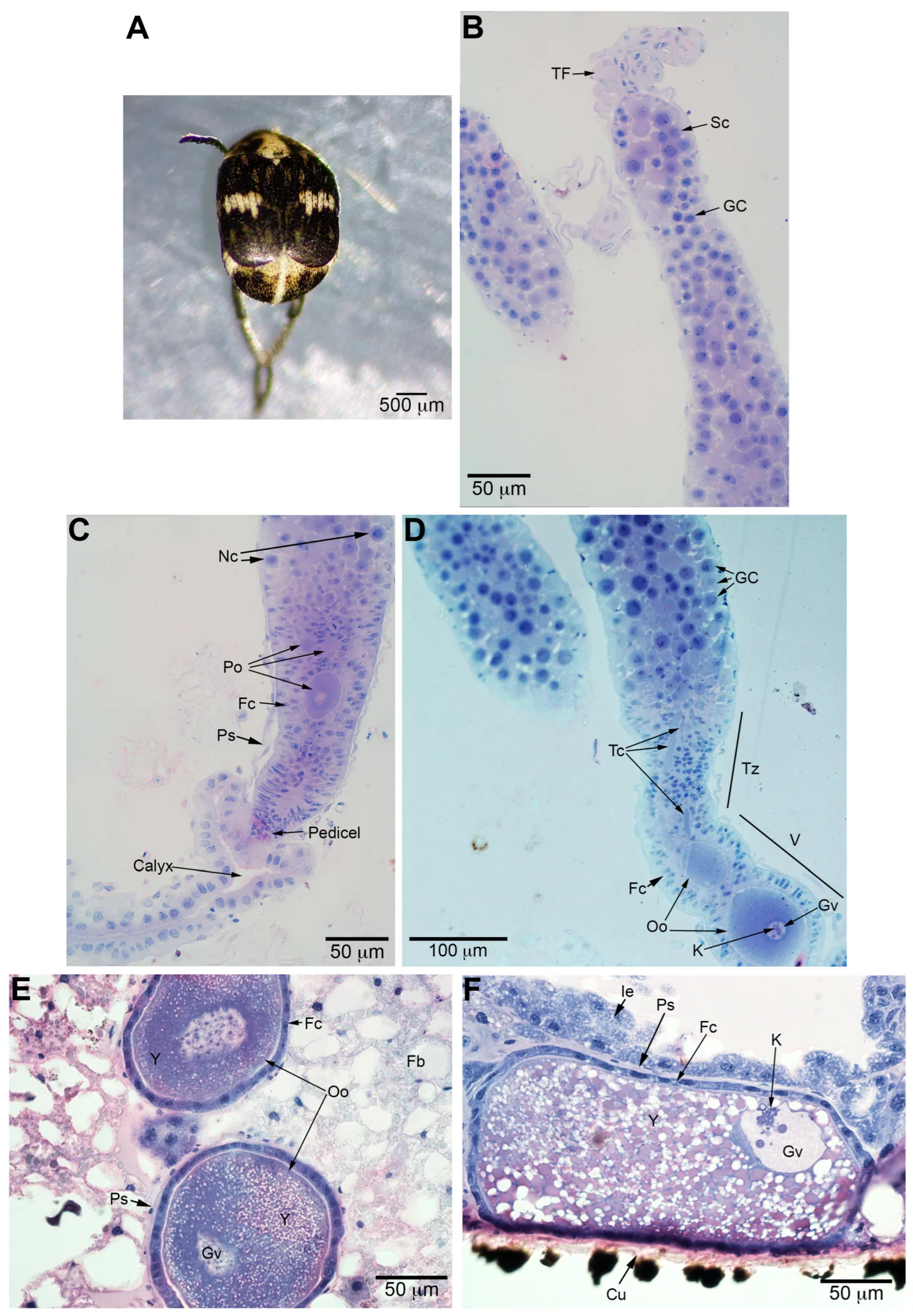
Morphology of the pharate-adult (24–25 days after egg laying) ovary of *Zabrotes subfasciatus*. (**A**) Stereomicroscope image of a female pharate adult. Light microscope images of dissected ovaries (**B**–**D**) and whole-body sections (**E**,**F**), shown in longitudinal and transverse orientations, respectively. TF, terminal filament; Sc, syncytial cells; GC, germ cells; Nc, nurse cell; Po, Pro-oocytes; Oo, oocytes; Fc, follicular cell; Tc, trophic cord; Tz, transition zone; V, vitellarium; Gv, germinal vesicle; K, karyosome; Y, yolk; Ps, peritoneal sheath; Ie, intestinal epithelium; Fb, fat body; Cu, cuticle.

In early pupae (20–22 days after egg laying), the ovarioles of female *Z. subfasciatus* remain relatively undifferentiated when compared with those of pharate adults, although important structural changes are already evident (Figure 5). At this stage, the ovariole is elongated and dominated by a conspicuous germarium containing densely packed germ cells and syncytial elements, indicating ongoing cellular proliferation and early differentiation. However, not clearly delimited vitellogenic regions are yet apparent, suggesting that the ovary is still in a previtellogenic state. By the pharate-adult stage (24–25 days after egg laying), the ovarioles display a marked increase in structural organization (Figure 6). The terminal filament becomes readily distinguishable (Figure 6B), and the germarium is more clearly defined. Proximal to this region, developing oocytes can be recognized and are associated with follicular cells and trophic cords (Figure 6D), revealing the onset of the telotrophic organization characteristic of the adult ovary. The duct-like structure observed in prior phases are now only a small pedicel connecting the vitellarium to the newly formed calyx (Figure 6C). The developing vitellarium contains some oocytes showing a conspicuous germinal vesicle, karyosome, and initial yolk deposition, indicating that vitellogenesis begins before adult emergence (Figure 6D–F).

### 3.4. Organization of the Adult Ovary of Zabrotes subfasciatus

**Figure 7:**
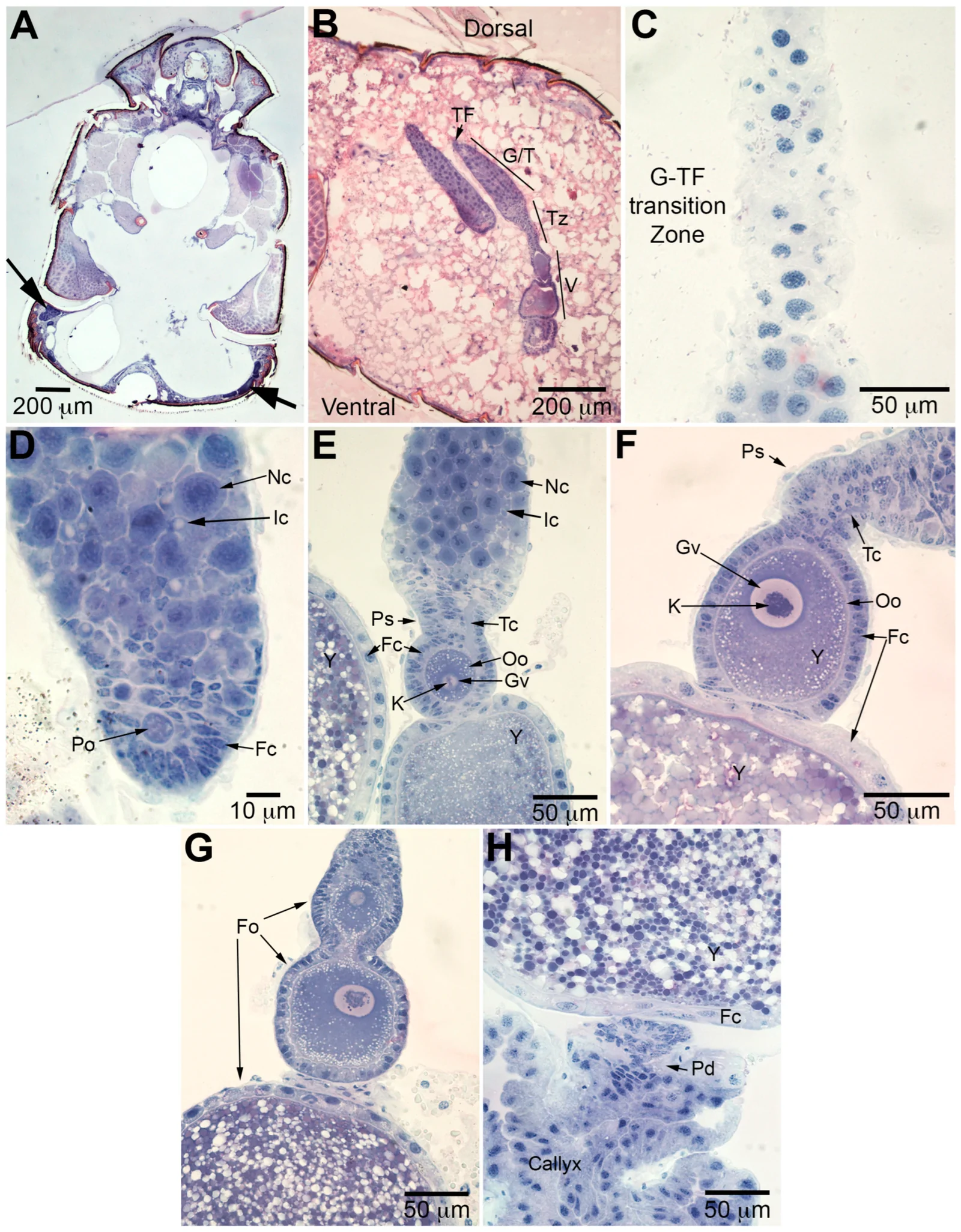
Morphology of the ovary in a four-day-old *Zabrotes subfasciatus* adult. Light microscope images of whole bodies (**A**,**B**), shown in frontal and longitudinal sections, respectively, and longitudinal sections of dissected ovaries (**C**–**H**). Arrows in (**A**) point to ovary pair localization. TF, terminal filament; G/T, germarium/tropharium; Tz, transition zone; G-TF, germarium-terminal filament; V, vitellarium; Nc, nurse cell; Ic, interstitial cell; Po, Pro-oocyte; Fc, follicular cell; Ps, peritoneal sheath; Tc, trophic cord; Oo, oocyte; Gv, germinal vesicle; K, karyosome; Y, yolk; Fo, follicles; Pd, Pedicel.

Light microscopy revealed that the ovary of a four-day-old *Z. subfasciatus* female maintained on beans in the presence of males, under optimal conditions for egg production, displays morphologically distinct ovariole regions—the terminal filament, germarium/tropharium, transition zone, and vitellarium—based on H&E and SEM criteria. Oocytes within the vitellarium are present at different developmental stages, from pro-oocytes (primary oocytes) to oocytes at advanced stage of differentiation, exhibiting progressive yolk accumulation (Figure 7).

**Figure 8:**
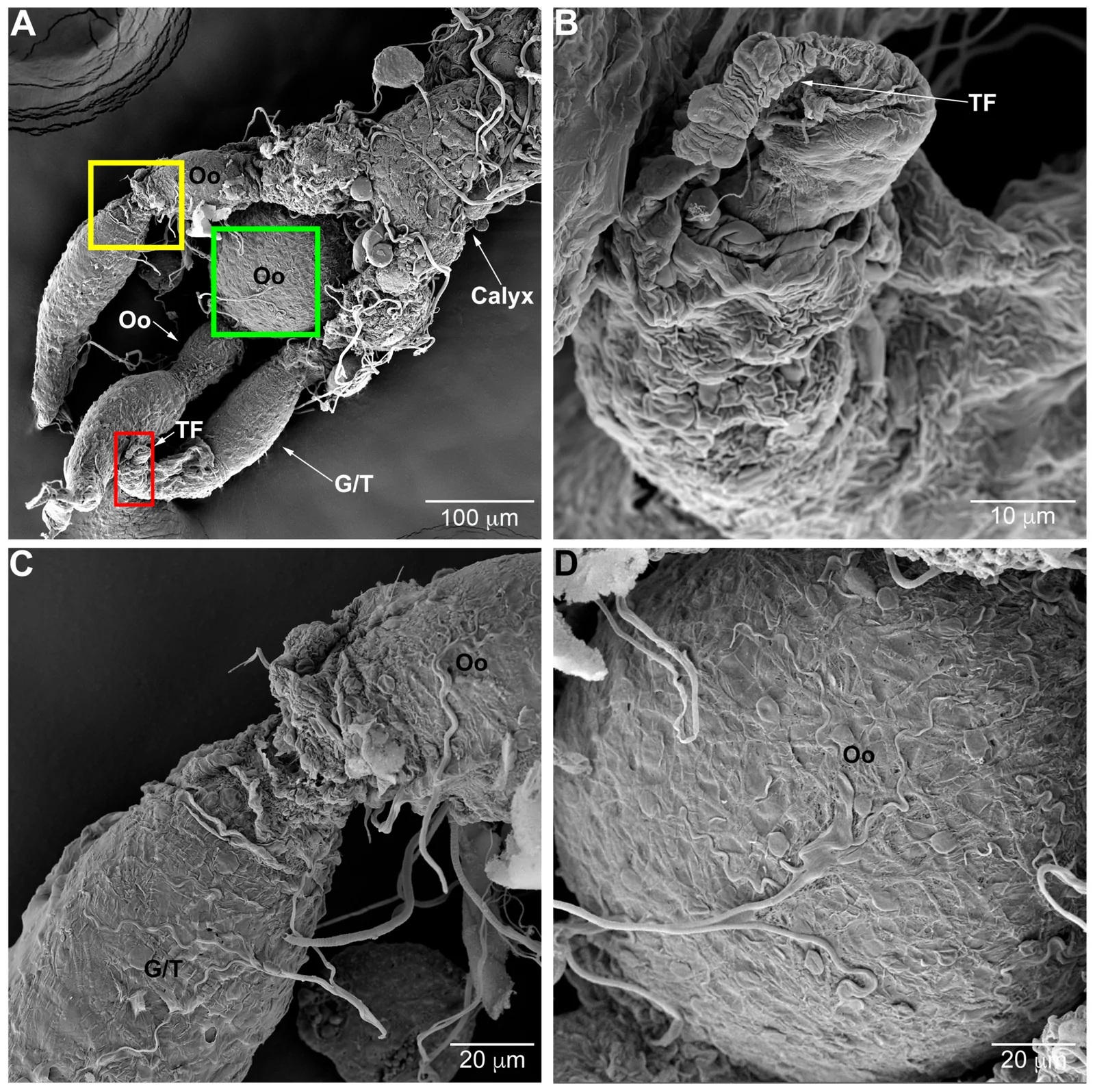
Structural organization of the ovary in a four-day-old *Zabrotes subfasciatus* adult. SEM images. (**A**) Whole ovary showing the three ovarioles, oocytes, germarium/tropharium and the calyx. (**B**) Terminal filament (red label in (**A**)). (**C**) Germarium-vitellarium transition zone (yellow label in (**A**)). (**D**) Oocyte within the vitellarium (green label in (**A**)). G/T, germarium/tropharium; Oo, oocyte; TF, terminal filament.

Scanning electron-microscope observations further distinguished the major ovariole regions, including the germarium/tropharium, the vitellarium with developing oocytes, and the adult calyx. Detailed images of the external morphology of the terminal filaments were also obtained (Figure 8).

### 3.5. Histological Organization of the Larval Testis of Zabrotes subfasciatus

**Figure 9:**
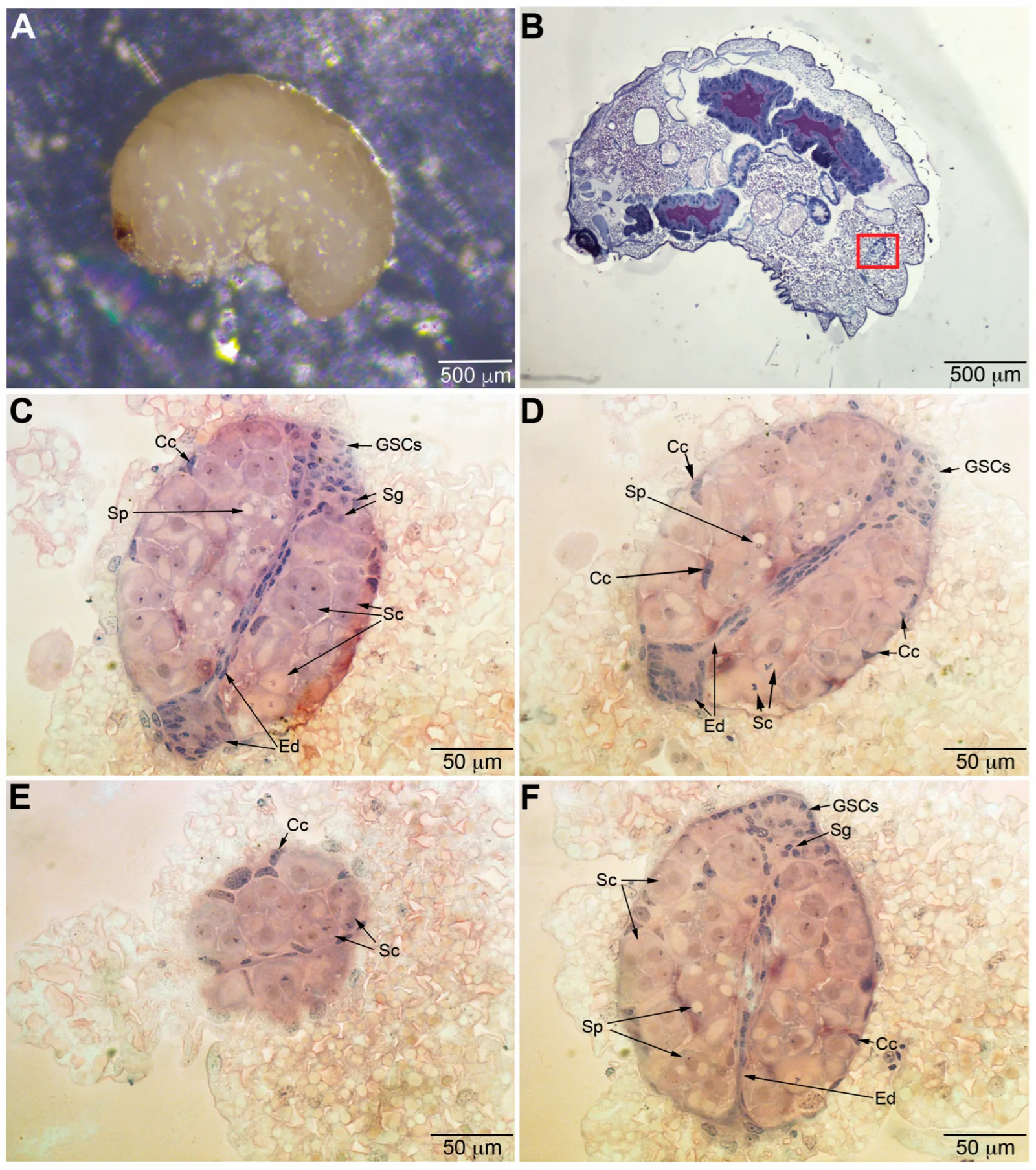
Morphology of the larval (14 days after egg laying) testes of *Zabrotes subfasciatus*. (**A**) Stereomicroscope image of a male larva. (**B**–**F**) Light microscope images. (**B**) Longitudinal section of a larval body highlighting the anatomical location of the testes (red label). (**C**–**F**) Sections of dissected testes. Cc, cyst cell; Sp, differentiating spermatids; GSCs, germline stem cells; Sg, spermatogonia; Sc, spermatocytes; Ed, efferent duct.

Light microscopy revealed that the testes of *Z. subfasciatus* larvae (14 days after oviposition) are in the posterior-middle region of the abdomen and are embedded within abundant fat body tissue (Figure 9). There are a pair of testes, each one formed by two follicles or lobes. The follicles are filled with numerous seminiferous cysts, each containing germ cells in the same stage of spermatogenesis (Figure 9C–F). The most distal region contains germ cells and clusters of spermatogonia, indicating active mitotic proliferation. Spermatogonia appear as small, densely stained cells with prominent nuclei. Their localization is characteristic of insect testes, where early germ-cell development occurs in defined germinal zones. More centrally, larger spermatocytes are observed. These cells exhibit increased cytoplasmic volume and larger well-stained or clear nuclei, consistent with progression toward meiosis. The presence of numerous spermatocytes demonstrates that germ-cell differentiation is well advanced during the larval stage. Several cysts contain spermatids, recognizable by their smaller size, condensed nuclei and a chromophobic, unstained circular area resembling a large vacuole, representing specialized cytoplasm area undergoing remodeling during spermiogenesis. Each germ-cell cyst is enclosed by elongated cyst cells, which surround groups of synchronously developing germ cells and provide structural and physiological support. Each testicular follicle also contains a centrally located lumen, consistent with an intrafollicular efferent duct that becomes enlarged distally (Figure 9C,D,F). Intrafollicular duct identity was inferred from its morphology and anatomical position in histological sections, consistent with descriptions from other coleopteran species [27,28,29].

### 3.6. Histological Organization of the Late Larval (Pharate Pupae) Testes of Zabrotes subfasciatus

**Figure 10:**
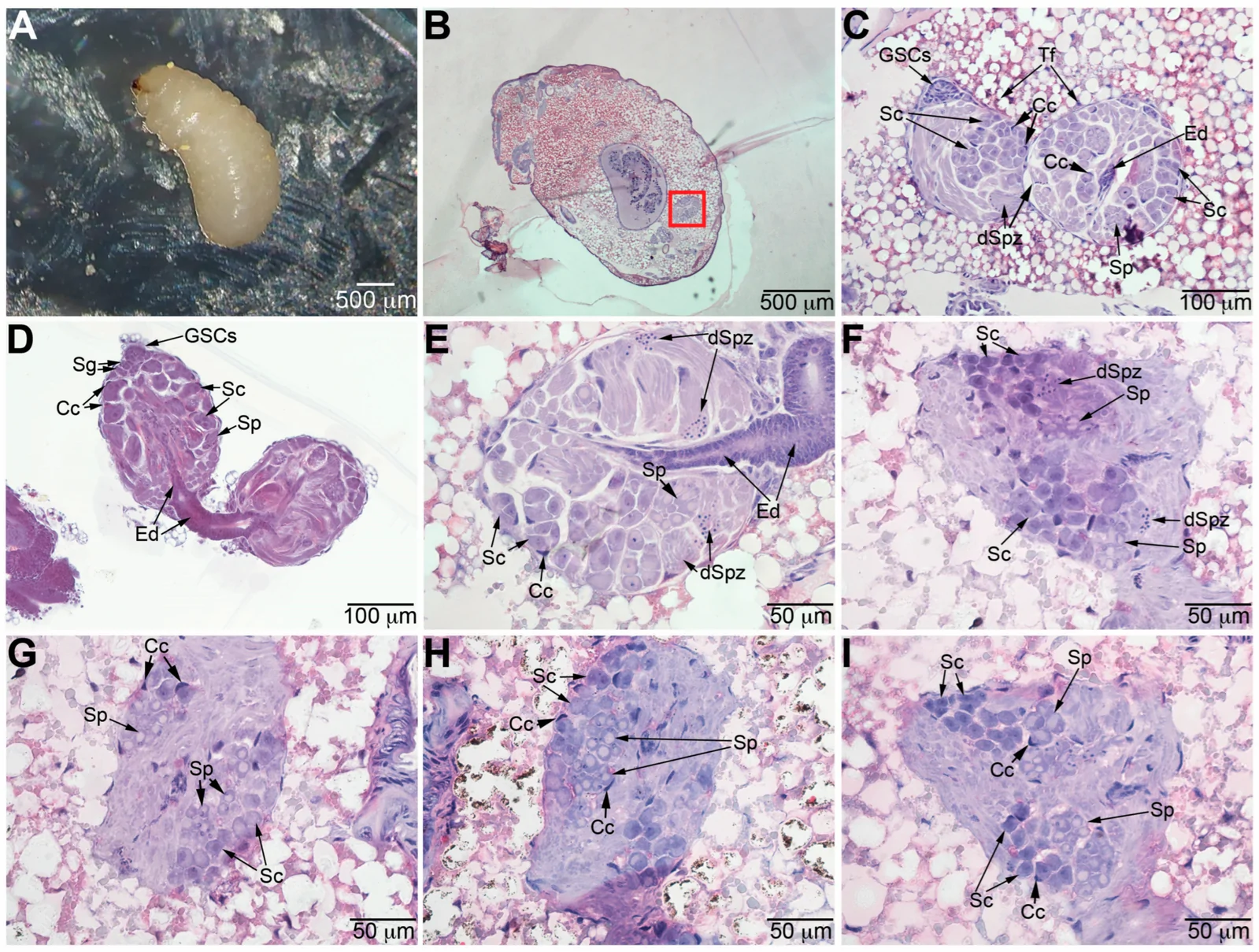
Morphology of the pharate-pupal (20–22 days after egg laying) testes of *Zabrotes subfasciatus*. (**A**) Stereomicroscope image of a male pharate pupa. (**B**–**I**) Light microscope images. (**B**) Longitudinal section of a pharate-pupal body highlighting the anatomical location of the testes (red label). (**C**–**I**) Magnified views of distinct sections from the red-labelled area indicated in (**B**), though in (**C**,**D**) follicle (lobe) pairs are shown. GSCs, germline stem cells; Sc, spermatocytes; Cc, cyst cell; Tf, testicular follicles; Sp, differentiating spermatids; Ed, efferent duct (intrafollicular and extrafollicular portions are seen in (**D**,**E**); Sg, spermatogonia; dSpz, differentiating spermatozoa.

Compared with early larval testes, pharate-pupal testes appear enlarged in our histological sections and exhibit a qualitatively higher degree of differentiation (Figure 10). At this stage, numerous densely packed differentiating spermatozoa are readily observed within the testicular follicles (Figure 10E,F), consistent with advanced spermiogenesis. Concomitantly, the proportion of early germ-cell stages is markedly reduced, whereas post-meiotic cells, particularly spermatids and differentiating spermatozoa, predominate. The organization of the germ-cell cysts reflects progressive testicular maturation during metamorphosis, characterized by active spermiogenesis and the accumulation of differentiating spermatozoa. The predominance of late spermatogenic stages, together with the relative scarcity of proliferative germ cells, indicates that spermatogenesis is well advanced by the pharate-pupal stage. Although pharate-pupal testes appear larger than larval testes, this observation remains qualitative because testis volume was not measured.

### 3.7. Histological Organization of the Early Pupal Testes of Zabrotes subfasciatus

**Figure 11:**
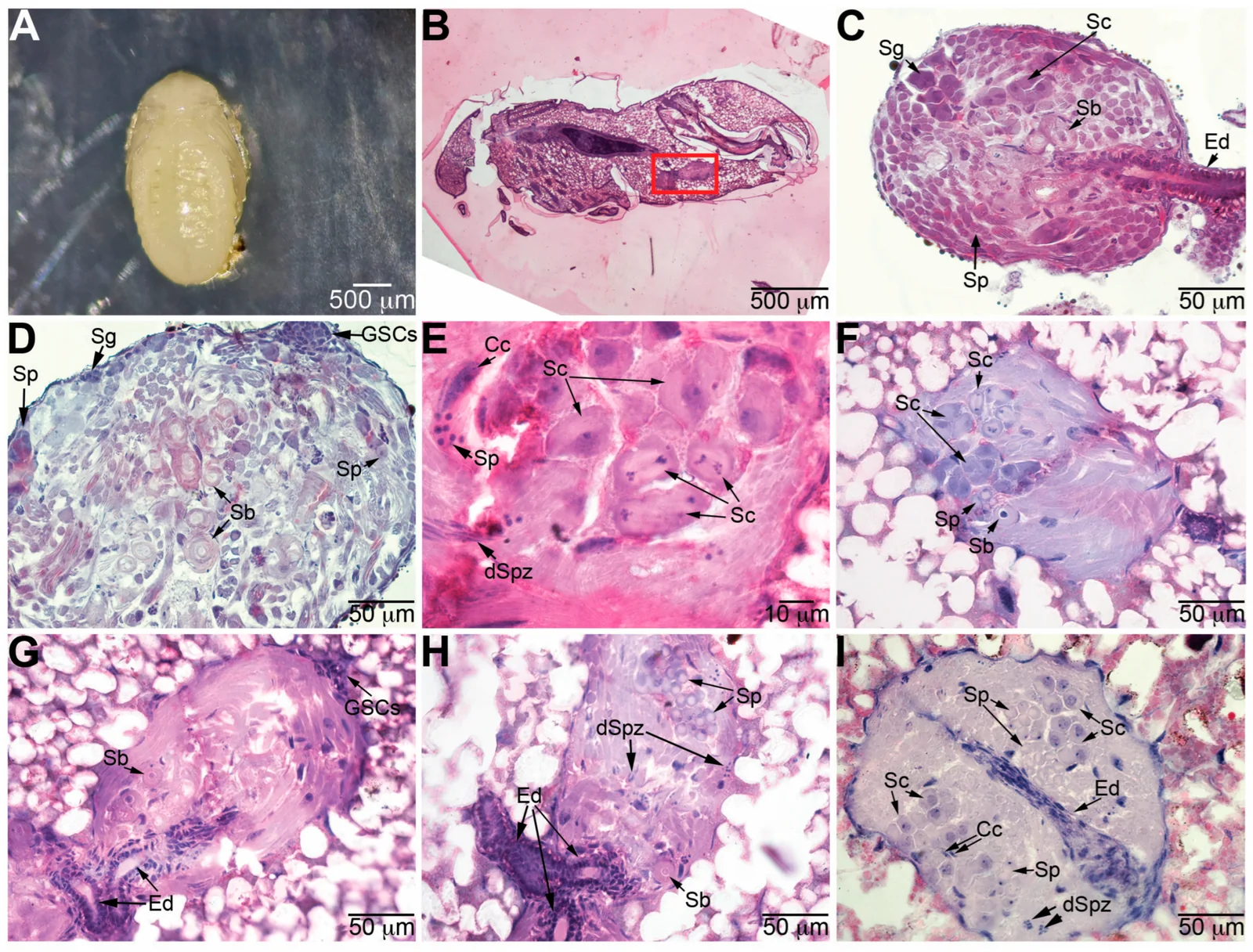
Morphology of the pupal (20–22 days after egg laying) testes of *Zabrotes subfasciatus*. (**A**) Stereomicroscope image of a male pupa. (**B**–**I**) Light microscope images. Whole body (**B**) and dissected testes (**C**,**D**). (**B**) Longitudinal section of a pupal body highlighting the anatomical location of the testes (red label). (**C**–**I**) magnified views of distinct sections from the red-labelled area indicated in (**B**). Cc, cyst cell; Sp, spermatids; GSCs, germline stem cells; Sg, spermatogonia; Sc, spermatocytes; Ed, efferent duct (intrafollicular and extrafollicular portions); dSpz, differentiating spermatozoa; Sb, sperm bundles.

Early pupal testes contain, in addition to germ stem cells and cysts representing different stages of spermatogenesis and spermiogenesis, numerous dense bundles of spermatozoa (Figure 11C,D,F,H). These conspicuous structures are frequently observed in association with the intrafollicular efferent duct, suggesting the progressive establishment of sperm transport pathways within the reproductive tract. The abundance of spermatids and differentiating spermatozoa indicates intense spermiogenesis and ongoing sperm maturation.

### 3.8. Histological Organization of the Pharate-Adult Testes of Zabrotes subfasciatus

**Figure 12:**
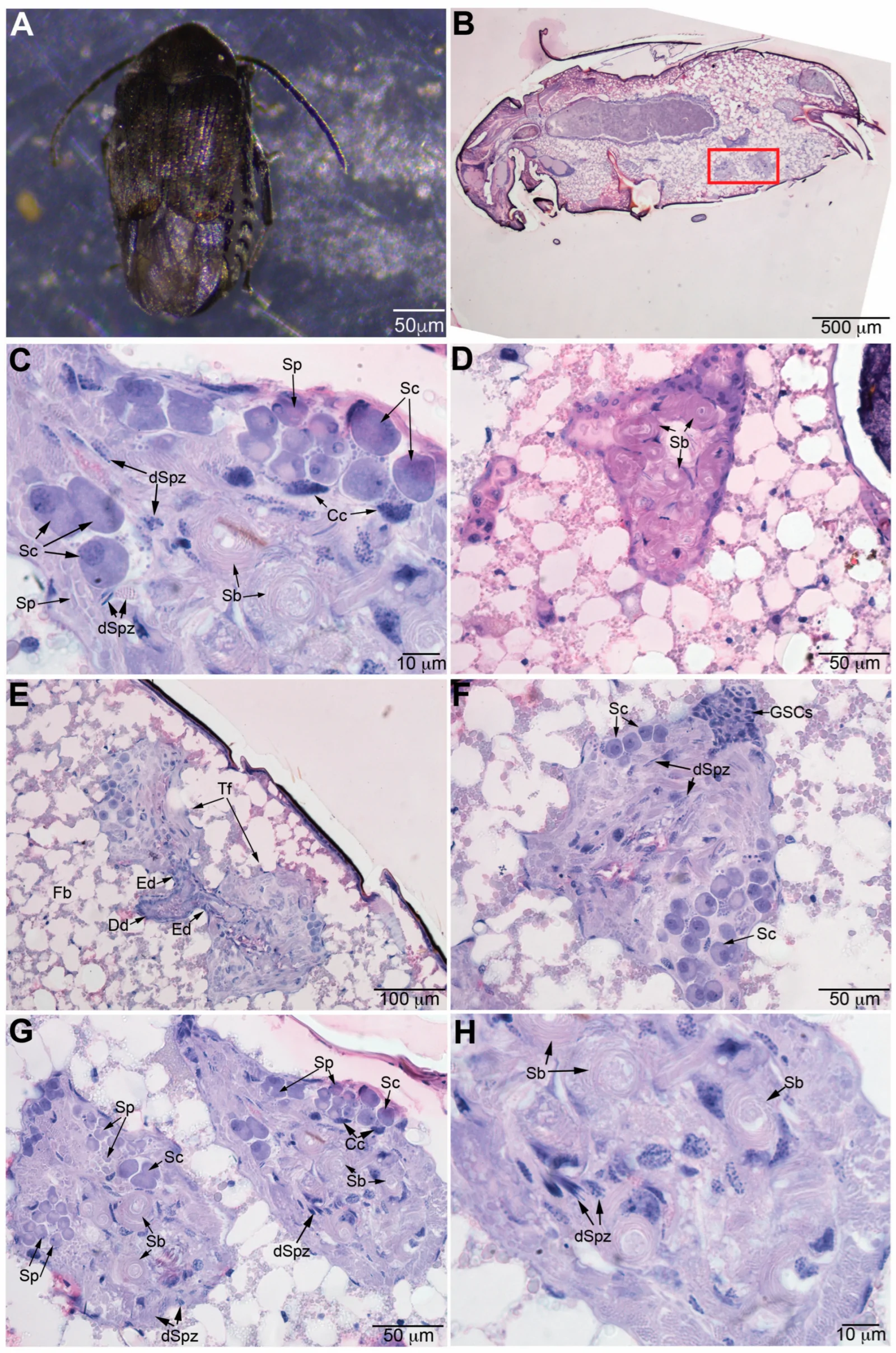
Morphology of the pharate-adult (24–25 days after egg laying) testes of *Zabrotes subfasciatus*. (**A**) Stereomicroscope image of a male pharate adult. (**B**–**H**) Light microscope images. (**B**) Longitudinal section of a pharate-adult body highlighting the anatomical location of the testes (red label). (**C**–**H**) magnified views of distinct sections from the red-labelled area indicated in (**B**). Cc, cyst cell; Sp, spermatids; GSCs, germline stem cells; Sc, spermatocytes; Ed, efferent duct; Dd, deferent duct; Tf, testicular follicles; dSpz, differentiating spermatozoa; Sb, sperm bundles; Fb, fat body.

Pharate-adult testes show coexisting spermatogenic stages (Figure 12), consistent with asynchronous development, although organ-wide quantification was not performed. In addition, small groups of cyst cells remain associated with same-stage groups of germ cells. The presence of multiple developmental stages within adjacent cysts indicates continuous and already highly active spermatogenesis (Figure 12).

### 3.9. Histological Organization of the Adult Testis of Zabrotes subfasciatus

**Figure 13:**
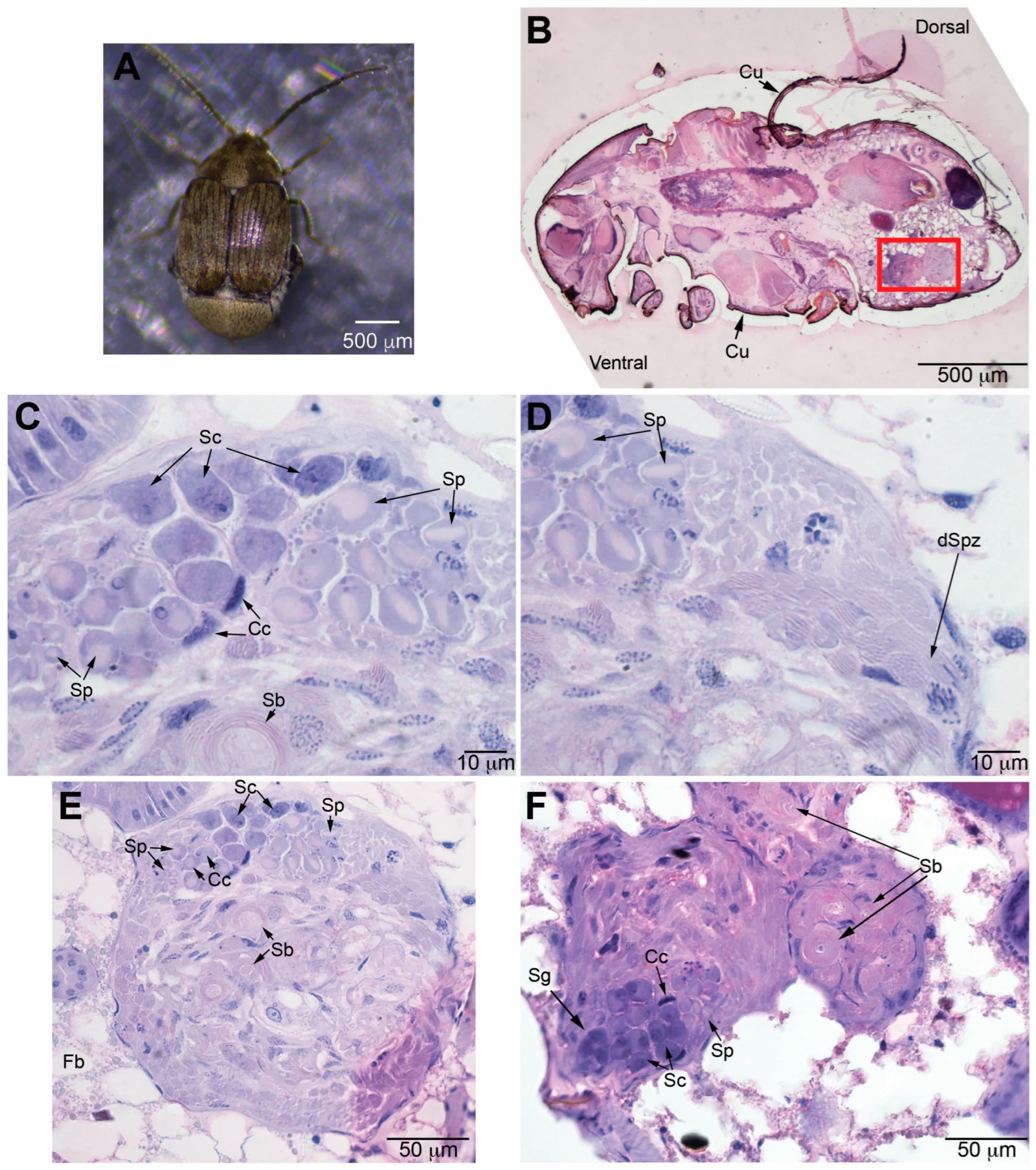
Morphology of the testes in a four-day-old *Zabrotes subfasciatus* adult. (**A**) Stereomicroscope image of an adult male. (**B**–**F**) Light microscope images. (**B**) Longitudinal section of an adult body highlighting the anatomical location of the testes (red label). (**C**–**F**), magnified views of distinct sections from the red-labelled area indicated in (**B**). Cu, cuticle; Sc, spermatocytes; Sp, differentiating spermatids; Cc, cyst cell; Sb, sperm bundles; Sg, spermatogonia; dSpz, differentiating spermatozoa; Fb, fat body.

Histological sections of four-day-old testes reveal fully differentiated gonads with coexisting spermatogenic stages and many sperm bundles; functional activity and sperm viability were not assayed. Different developmental stages of the germ line coexist within the testicular follicles, indicating continuous sperm production in the sexually mature male (Figure 13).

**Figure 14:**
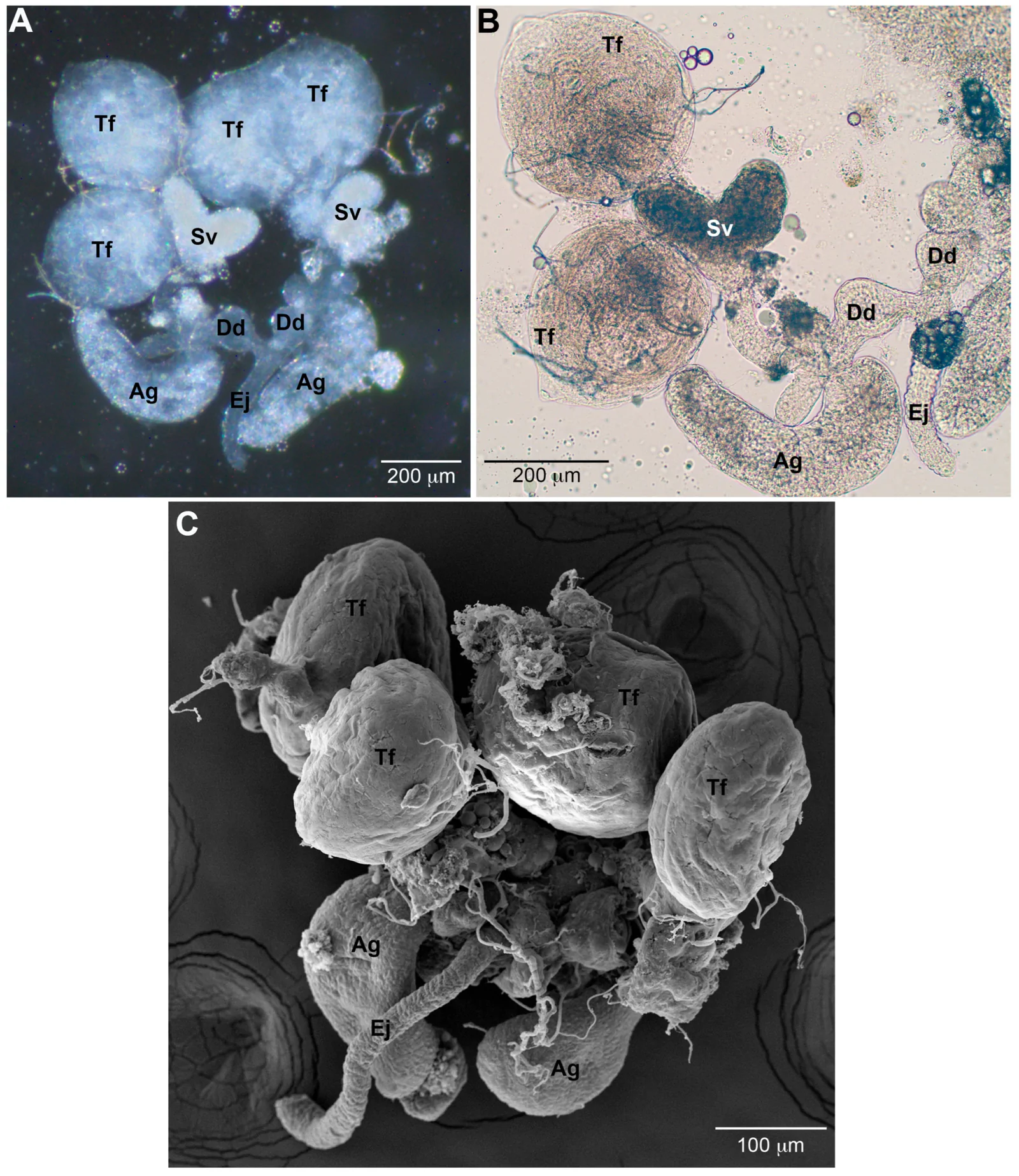
Structural organization of the reproductive system of a four-day-old *Zabrotes subfasciatus* adult male. Stereomicroscope (**A**), light microscope (**B**), and SEM (**C**) images of dissected testes and reproductive structures. Tf, testicular follicle; Sv, seminal vesicle; Dd, deferent duct; Ag, accessory gland; Ej, ejaculatory duct.

The male reproductive system of a four-day-old adult *Z. subfasciatus*, observed under stereomicroscope, exhibits a fully differentiated reproductive apparatus indicative of active sperm production, storage, and reproductive readiness. The paired testes are each composed of rounded follicles (lobes) that connect medially to the seminal vesicles, which likely function in the storage of sperm released from the follicles. Posteriorly, the reproductive tract continues through the deferent ducts and ejaculatory duct, while a pair of well-developed accessory glands is associated with its distal region (Figure 14A,B). Light microscopy confirms the bilobed organization of each testis and reveals densely packed cellular contents corresponding to germ cells at different stages of spermatogenesis, as well as the presence of sperm bundles, indicating intense spermatogenic activity and sperm accumulation (Figure 14B). Scanning electron microscopy further illustrates the three-dimensional organization of the reproductive system, highlighting the compact arrangement of the follicles, accessory glands, and ejaculatory duct, as well as the smooth to slightly folded surface of the follicular sheath (Figure 14C).

## 4. Discussion

Using stereomicroscopy, light microscopy, and scanning electron microscopy, we establish a stage-by-stage morphological framework for gonad maturation in *Z. subfasciatus*. Our analyses reveal that post-embryonic gonad development begins during larval life but reaches its principal structural and functional organization during late metamorphosis. This developmental trajectory supports the hypothesis that reproductive readiness in this capital-breeding seed beetle is established before adult emergence, rather than being deferred until adulthood. In females, the larval and pharate-pupal ovaries are characterized primarily by proliferative germaria and posterior duct-like regions, whereas the defining features of telotrophic meroistic ovarioles become evident during the pupal-to-pharate-adult transition. The differentiation of individualized oocytes, follicular cells, trophic cords, a distinct vitellarium, and the onset of yolk deposition before adult emergence indicates that ovarian maturation is already underway during the pharate-adult stage. This interpretation is further supported by the establishment of a clear germarium-vitellarium transition, follicular encapsulation of developing oocytes, and fully regionalized adult ovarioles. Although we did not employ molecular or ultrastructural approaches to confirm yolk identity or the ultrastructure of trophic cords, the observed morphological changes strongly indicate the onset of ovarian function before adult emergence. These findings are consistent with previous evidence showing that vitellogenesis in *Z. subfasciatus* begins during late metamorphosis and that subsequent ovarian activation is modulated by host seeds and male presence after adult emergence [9].

The ovarian sequence described here clarifies how the adult telotrophic architecture is assembled during metamorphosis. In telotrophic meroistic ovarioles, nurse-cell and germ stem cell derivatives remain concentrated in the tropharium, while developing oocytes remain connected to this region by trophic cords and are progressively enclosed by follicular cells [30]. In *Z. subfasciatus*, this organization is only weakly defined in larvae and pupae, but becomes evident in pharate adults, when the transition between tropharium and vitellarium can be recognized. Thus, the pupal-to-pharate-adult interval appears to be the critical morphogenetic window for establishing the functional compartments of the ovary. The convergence of the posterior duct-like regions of the three ovarioles into a common terminal structure during pharate-pupal and pupal development likely represents formation of the future calyx. Comparable differentiation of ovariole pedicels and associated duct systems has been described in insects with telotrophic ovaries, including *Triatoma infestans* and *Rhodnius prolixus* [31,32,33], as well as in the curculionid *Hyperodes bonariensis* [34]. In *Z. subfasciatus*, completing this ductal integration before emergence may facilitate the rapid transition from ovarian maturation to effective oviposition.

Male gonad development follows a similarly early trajectory, although it appears to progress more continuously than ovarian maturation. The analyzed larval testis (14 days after egg laying) is not an undifferentiated gonad; rather, it already contains germ stem cells, spermatogonia, spermatocytes, spermatids, cyst cells, and intrafollicular efferent ducts. The presence of spermatids indicates that meiotic divisions have already occurred and that spermiogenesis begins before pupation (as reported for example for the bloodsucking horn fly, *Haematobia irritans*, [35]). In these cells, the condensed nucleus is associated with a chromophobic cytoplasmic region that typically corresponds to the nebenkern. This spherical structure, which may appear histologically similar to a large vacuole, derives from the aggregation and fusion of the spermatid mitochondrial complement and is therefore enriched in mitochondrial membrane lipids. Its composition likely explains its weak staining with the H&E used here [36,37]. Nevertheless, while the H&E morphology and cyst architecture observed in this study strongly support the presence of post-meiotic cells in larvae, unequivocal discrimination between late spermatocytes and early spermatids will require ultrastructural analysis or stage-specific marker validation. Taken together, these observations indicate that the basic architecture of the male reproductive tract is established during larval life, although full functional integration is completed later. The close association between testes and fat body tissue further supports the high energetic demands of germ-cell proliferation and differentiation. Overall, the larval testis exhibits the cystic organization characteristic of Coleoptera (e.g., [38]) and demonstrates that male gametogenesis is already advanced before metamorphosis.

During the pharate-pupal and pupal stages, testicular maturation becomes increasingly pronounced. The abundance of spermatids, differentiating spermatozoa, and dense sperm bundles indicates that spermiogenesis predominates during this interval, while the simultaneous presence of meiotic cells, post-meiotic spermatids, and sperm bundles shows that multiple waves of spermatogenesis occur concurrently. Together, the predominance of late spermatogenic stages and the relative scarcity of early germ-cell stages indicate that testicular maturation is nearing completion before adult emergence. This pattern resembles the precocious development reported for other insects, in which substantial sperm production occurs before adulthood (e.g., [39]), and is likely adaptive in *Z. subfasciatus*, whose adults are short-lived and initiate reproductive activity soon after emergence [9,40,41]. By the pharate-adult stage, testes still contain multiple spermatogenic stages simultaneously, consistent with asynchronous activity, although lobe-wide staging was not quantified. In four-day-old adult males, the reproductive system is fully differentiated, including compact testicular follicles, seminal vesicles, deferent ducts, accessory glands, and the ejaculatory duct. The persistence of early germ cells alongside abundant sperm bundles indicates that sperm production continues after emergence, thereby maintaining a continuous sperm supply during adult reproductive life.

Together, the female and male data indicate that metamorphosis in *Z. subfasciatus* does more than enlarge pre-existing gonadal structures: it reorganizes proliferative larval organs into compartmentalized reproductive systems that are largely prepared for function by adult emergence [9]. This developmental schedule fits the biology of Bruchinae, whose larvae develop within seeds and whose adults frequently rely on larval reserves to support reproduction [1]. In addition to most Bruchinae, this dependence of reproductive development on nutritional inputs acquired during the larval stage has also been reported in other pest Coleoptera, including the agricultural and turf pest *Cyclocephala barrerai*, the red flour beetle *Tribolium castaneum*, and the yellow mealworm beetle *Tenebrio molitor*, in which gonadal development has been shown to depend on larval nutritional conditions [42,43]. Under this life-history strategy, early gonad maturation may reduce the delay between emergence, mating, and oviposition, thereby increasing reproductive efficiency in a pest species with a short adult lifespan [40].

## 5. Conclusions

**Figure 15:**
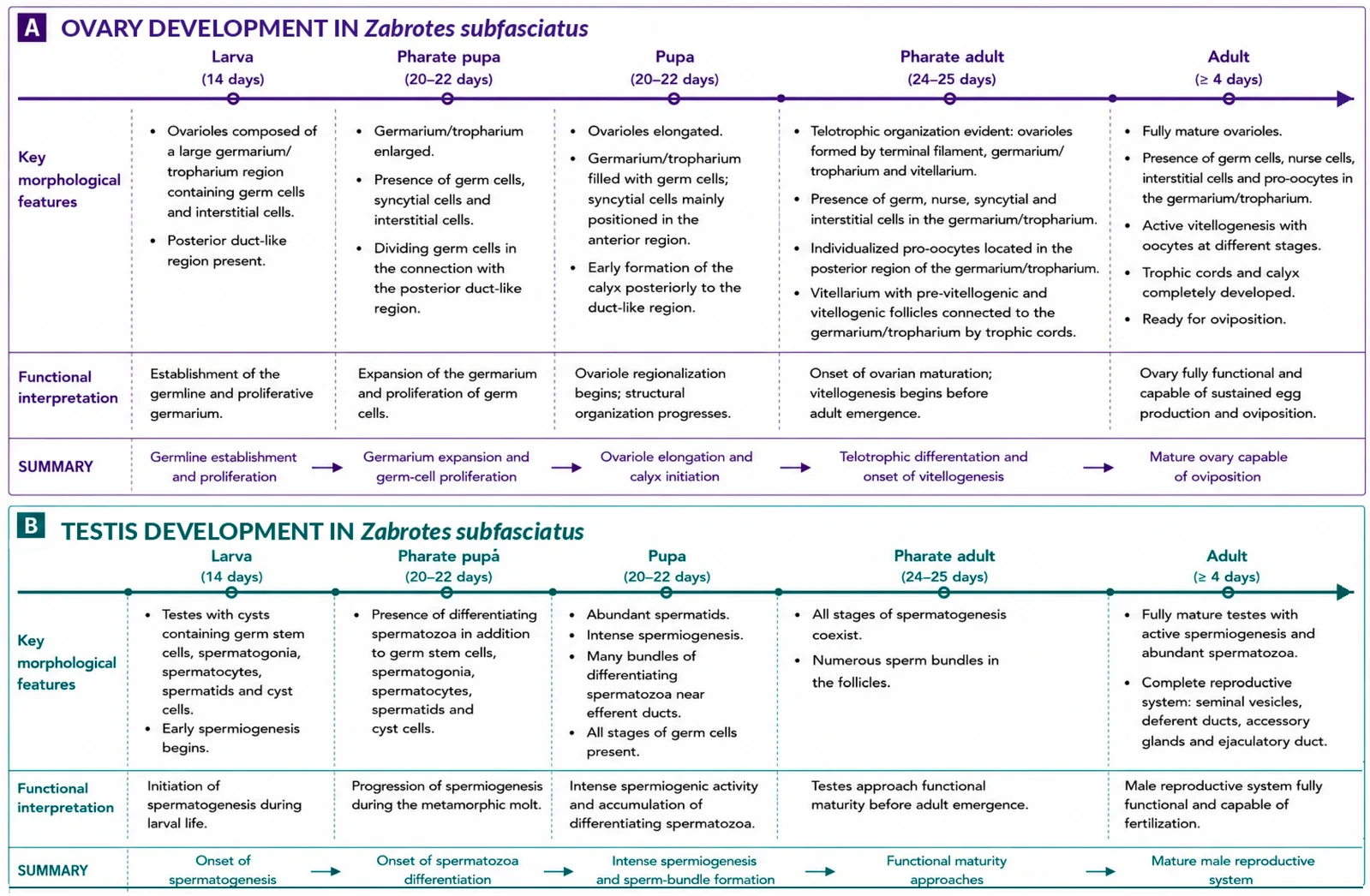
Timeline of post-embryonic gonad development in *Zabrotes subfasciatus*. The upper timeline summarizes the major morphological events during ovary development, whereas the lower timeline shows the corresponding stages of testis development. Developmental stages are arranged chronologically according to age after egg laying: larva (14 days), pharate pupa (20–22 days), pupa (20–22 days in females; early and late pupal stages in males), pharate adult (24–25 days), and adult (4 days after emergence). In females, (**A**), ovarian development progresses from proliferative germaria and undifferentiated ovarioles to the establishment of a telotrophic meroistic organization, with oocyte differentiation, formation of follicular cells and trophic cords, and the onset of vitellogenesis before adult emergence. In males, (**B**), testicular development is characterized by progressive spermatogenesis, increasing abundance of spermatids and sperm bundles, differentiation of the reproductive tract, and completion of reproductive maturation shortly after adult emergence. Days indicate the approximate age after egg laying under the rearing conditions used in this study.

This study demonstrates that gonad development in *Z. subfasciatus* is initiated during larval life and becomes functionally organized mainly during late metamorphosis. In females, the transformation from proliferative larval ovarioles to an adult-like telotrophic meroistic organization occurs chiefly between the pupal and pharate-adult stages, when germarial and vitellogenic regions become distinguishable, follicular cells and trophic cords appear, and vitellogenesis begins before adult emergence. In males, testicular differentiation is also advanced before emergence, with larval testes already containing multiple germ-cell stages and later stages showing intense spermiogenesis, sperm-bundle accumulation, and differentiation of the reproductive tract (Figure 15). Together, these findings support the hypothesis that reproductive readiness in this capital-breeding seed beetle is developmentally anticipated rather than delayed until adult life. By integrating female and male gonad ontogeny across post-embryonic development, this work provides a morphological framework for understanding the rapid onset of reproduction in newly emerged adults and establishes a basis for future studies on endocrine regulation, reproductive ecology, and strategies aimed at disrupting reproduction in this economically important pest.

## Data Availability

The original contributions presented in this study are included in the article. Further inquiries can be directed to the corresponding author.
